# Universal gene co-expression network reveals receptor-like protein genes involved in broad-spectrum resistance in pepper (*Capsicum annuum* L.)

**DOI:** 10.1093/hr/uhab003

**Published:** 2022-01-19

**Authors:** Won-Hee Kang, Junesung Lee, Namjin Koo, Ji-Su Kwon, Boseul Park, Yong-Min Kim, Seon-In Yeom

**Affiliations:** 1 Institute of Agriculture & Life Science, Gyeongsang National University, 501, Jinju-daero, Gajwa-dong, Jinju, 52828, Republic of Korea; 2 Department of Horticulture, Division of Applied Life Science (BK21 four), Gyeongsang National University, 501, Jinju-daero, Gajwa-dong, Jinju, 52828, Republic of Korea; 3 Korean Bioinformation Center, Korea Research Institute of Bioscience and Biotechnology, 125, Gwahak-ro, Yuseong-gu, Daejeon, 34141, Republic of Korea; 4 Genome Engineering Research Center, Korea Research Institute of Bioscience and Biotechnology, 125, Gwahak-ro, Yuseong-gu, Daejeon, 34141, Republic of Korea

## Abstract

Receptor-like proteins (RLPs) on plant cells have been implicated in immune responses and developmental processes. Although hundreds of *RLP* genes have been identified in plants, only a few RLPs have been functionally characterized in a limited number of plant species. Here, we identified *RLPs* in the pepper (*Capsicum annuum*) genome and performed comparative transcriptomics coupled with the analysis of conserved gene co-expression networks (GCNs) to reveal the role of core RLP regulators in pepper–pathogen interactions. A total of 102 RNA-seq datasets of pepper plants infected with four pathogens were used to construct CaRLP-targeted GCNs (CaRLP-GCNs). Resistance-responsive CaRLP-GCNs were merged to construct a universal GCN. Fourteen hub *CaRLPs*, tightly connected with defense-related gene clusters, were identified in eight modules. Based on the CaRLP-GCNs, we evaluated whether hub *CaRLPs* in the universal GCN are involved in the biotic stress response. Of the nine hub *CaRLPs* tested by virus-induced gene silencing, three genes (*CaRLP264*, *CaRLP277*, and *CaRLP351*) showed defense suppression with less hypersensitive response-like cell death in race-specific and non-host resistance response to viruses and bacteria, respectively, and consistently enhanced susceptibility to *Ralstonia solanacearum* and/or *Phytophthora capsici*. These data suggest that key *CaRLPs* are involved in the defense response to multiple biotic stresses and can be used to engineer a plant with broad-spectrum resistance. Together, our data show that generating a universal GCN using comprehensive transcriptome datasets can provide important clues to uncover genes involved in various biological processes.

## Introduction

Plants use extra- and intracellular immune signaling to protect themselves against pathogens [1, 2]. The first layer of plant immunity, known as pattern-triggered immunity, is activated following the perception of pathogen- or microbe-associated molecular patterns (PAMPs or MAMPs) by plant cell surface-localized pattern recognition receptors (PRRs). PRRs sense diverse pathogens, including bacteria, fungi, oomycetes and parasitic plants, and are involved in complex immune signaling networks [3]. Recently, plant PRRs have been successfully used to confer broad-spectrum resistance in potato (*Solanum tuberosum*) [4], *Nicotiana benthamiana,* and tomato (*Solanum lycopersicum*) [5] and have been considered to confer broad-spectrum disease resistance in other crops.

Plant PRRs are classified into two main classes depending on their cytoplasmic kinase domains: receptor-like kinases (RLKs) and receptor-like proteins (RLPs). RLKs contain an extracellular domain, a single transmembrane domain, and a cytoplasmic kinase domain, whereas RLPs lack the cytoplasmic kinase domain but carry a short cytoplasmic tail. RLPs play crucial roles in plant immunity against pathogens. The first RLPs, designated as *Cf* genes, were identified in tomato, which imparted resistance to *Cladosporium fulvum* isolates [6–9]. Since then, several RLPs have been shown to play roles in plant defense, mostly in Solanaceous plants and *Arabidopsis thaliana* [4, 10–18]. RLPs constitutively interact with the RLK suppressor of BIR1–1/EVERSHED (SOBIR1/EVR, hereafter SOBIR1), providing a kinase domain for intracellular signaling [2,3]. RLK BRI1-associated receptor kinase1/somatic embryogenesis kinase (BAK1/SERK3, hereafter BAK1) is also required for RLP/SOBIR1 complex on ligand recognition by RLP-mediated immunity [2, 3, 19]. In addition, RLPs are involved in plant development [20–23]. A number of genes encoding RLPs have been identified with the completion of the plant genome project [24–29]; however, relatively fewer genes have been functionally characterized to date.

Based on the recent advances in sequencing technology, along with the decline in the cost of sequencing, RNA-seq has been widely utilized in plants, producing massive amounts of data. However, identifying and manipulating information of interest from large integrated datasets remain challenging. Because functionally associated genes often show transcriptional co-regulation, gene co-expression networks (GCNs) present an important resource to identify novel genes within a given biological process-regulating module. Thus, the analysis of GCNs can be a powerful approach to predict gene functions and isolate modules involved in specific biological processes across large-scale gene expression data [30–33]. In recent years, GCN analysis has been successfully used to identify stress-responsive genes in plants [34–36]. Additionally, several research groups recently performed comparative and combined analyses of GCNs in time-series experiments conducted under various conditions and with multiple treatments, across different species and kingdoms [37–41]. These studies were used to identify hub genes and infer their roles in biological processes. However, GCN studies in crop plants are less well investigated than some other model species because of their extreme complexity and limited resources.

Chili pepper (*Capsicum* spp.), a member of the Solanaceae family, is an important vegetable crop worldwide. However, pepper production is threatened by pathogens such as fungi, bacteria, viruses, insects, and nematodes. The development of pathogen-resistant cultivars is one of the best approaches to control infection in pepper. Although multiple reference genomes and transcriptome datasets of pepper have been published recently [42–45], the molecular mechanism underlying plant immunity remains unclear. Therefore, comprehensive transcriptome data could provide important clues to identify and characterize genes involved in plant defense. In the present study, we identified 438 RLP genes in the chili pepper genome using phylogenetic and comparative transcriptomic analyses of 102 RNA-seq datasets of chili pepper plants challenged with four different pathogens. In addition, we constructed CaRLP-targeted GCNs (CaRLP-GCN) using comprehensive RNA-seq datasets and merged the resistant-responsive GCNs to develop a universal CaRLP-GCN. Using this GCN, we identified 14 putative RLP hub genes belonging to eight modules. Gene knock-down analysis of three *CaRLPs* (*CaRLP264*, *CaRLP277*, and *CaRLP351*) validated the broad immune response to pathogens. Silencing each of the three *CaRLPs* significantly reduced broad-spectrum resistance against viruses, bacteria, and oomycetes. Overall, this study demonstrates the successful characterization of novel genes by constructing a universal GCN from large RNA-seq datasets and provides key insights into broad-spectrum resistance in plants.

## Results

### Genome-wide identification and classification of RLPs in the pepper genome

**Figure 1:**
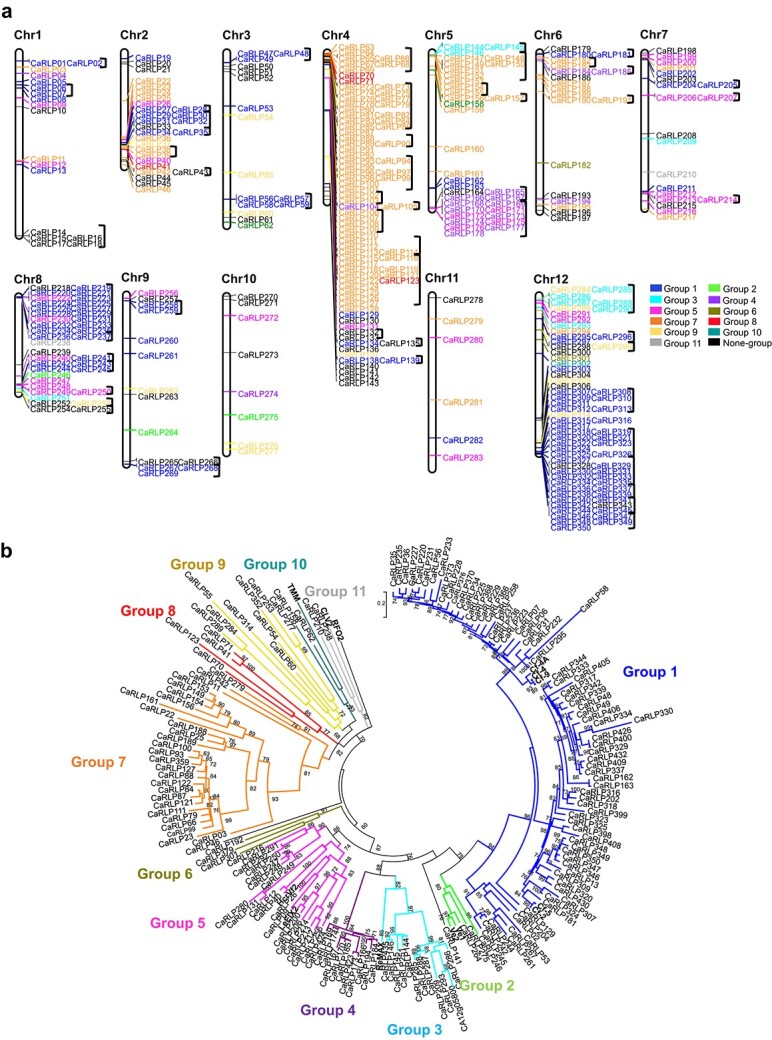
**Phylogenetic analysis and chromosomal locations of *CaRLPs*.** (a) Physical location of *CaRLP* genes on pepper chromosomes. Three hundred fifty *CaRLPs* were located on 12 chromosomes, while 88 *CaRLPs* were assigned to pepper scaffolds. The chromosome’s names are indicated at the top of each chromosome. *CaRLPs* are colored according to their phylogenetic group. Black square brackets on the right side of gene IDs indicate the physical gene clusters of CaRLPs on chromosomes**.** (b) Phylogenetic tree of CaRLPs constructed using the maximum-likelihood method of PhyML. Bootstrap values over 60 are indicated above the branches.

We identified 438 RLP-encoding genes in *Capsicum annuum (C. annuum)* genome by excluding redundant sequences and genes encoding NB-ARC or kinase domain-containing proteins and by validating the RLP structure (see Materials and Methods for details). The RLP-encoding genes were renamed according to their chromosomal positions (Fig. 1a and Supplementary Table S1). Details of the *CaRLP* genes are summarized in Supplementary Table S1. Phylogenetic analysis and sequence similarity-based clustering methods [26, 46] classified 364 of 438 CaRLPs into 11 groups; the remaining 74 CaRLPs were not classified into any group and were defined as singletons (Fig. 1b and Supplementary Table S1). Most of the RLPs were assigned to two groups (1 and 7). Group 1 was the largest group with 153 genes, including tomato *SlCf* genes and their homologs in pepper. Group 7 was the second largest group with 118 *CaRLP* genes, without known genes. Next, to explore the evolutionary relationships of CaRLPs with *Solanum lycopercisum* RLPs (SlRLPs) [26] and *A. thaliana* RLPs (AtRLPs) [24], we conducted a phylogenetic analysis of the amino acid sequence of the conserved C3-D domain of these RLPs (Supplementary Fig. S1). Most of the CaRLPs clustered together with SlRLPs, whereas most of the AtRLPs were grouped separately, forming two *Arabidopsis*-specific clades (Supplementary Fig. S1). These results suggest that the *CaRLP* gene family had undergone an expansion after divergence from the common ancestor of *Arabidopsis* and Solanaceae species.

### Chromosomal location, physical cluster, and conserved motif analyses

Of the 438 *CaRLP* genes identified in this study, 350 mapped to 12 chromosomes, while 88 were assigned to unmapped scaffolds (Fig. 1a). Most of the *CaRLPs* belonging to the same phylogenetic group were closely clustered on a given chromosome. Next, we performed physical cluster analysis to thoroughly investigate the chromosomal distribution of *CaRLPs*. Fifty-four clusters were identified on pepper chromosomes containing 227 genes (Fig. 1a and Supplementary Table S2). Each of these clusters spanned a physical distance of 0.7–885 kb. Large clusters (>200 kb) were located on chromosomes 1, 4, 5, 8, and 12, and no clusters were identified on chromosomes 10 and 11. Of all the *CaRLPs* in each group, those with large numbers (groups 1 and 7) formed mostly physical clusters. To better understand the *CaRLP* gene family, we examined conserved motifs in CaRLP proteins. Twenty distinct motifs were predicted among all 438 CaRLPs and known RLPs (Supplementary Fig. S2 and Supplementary Table S3). Most of the motifs encoded the leucine-rich repeat (LRR) domain, while motif 9 encoded the transmembrane region. Most of the closely related genes in the same phylogenetic group exhibited common motif compositions. Taken together, these data indicate that CaRLPs belonging to the same phylogenetic group share conserved motifs, similar protein domain compositions, and similar chromosomal locations.

### Expression analysis of *CaRLPs* in response to biotic stresses

RLPs perform crucial roles in plant disease resistance. However, little is known about the possible function of CaRLPs in the defense response. To further understand the role of *CaRLP* genes in plant defense, we investigated the expression patterns of *CaRLPs* showing differential expression between uninoculated (control) and pathogen-challenged pepper plants; these genes are hereafter referred to as differentially expressed *CaRLP* genes (*CaRLP*-DEGs). These DEGs were obtained from 63 previously published RNA-seq datasets of pepper plants infected with three different viruses, *Tobacco mosaic virus* (TMV) pathotype P0 (TMV-P0), TMV pathotype P2 (TMV-P2), and *Pepper mottle virus* (PepMoV) [45, 47]. In addition, to determine the changes in *CaRLP* expression at an early stage of oomycete infection, we generated six-timepoint RNA-seq datasets from three biological replicates of *P. capsici*-inoculated and control pepper plants (Supplementary Table S4). Thus, we examined a total of 102 RNA-seq datasets to analyze the expression of *CaRLPs* (Supplementary Table S5). Pepper accession “CM334,” which was used for RNA-seq analysis in this study, has different resistance responses—hypersensitive response (HR) against TMV-P0 and *P. capsici* and extreme resistance to PepMoV—but is susceptible to TMV-P2 [47–50]. To validate different plant responses to each pathogen, principal component analysis (PCA) was performed and showed clear variations in response dynamics of transcriptomes between samples (Supplementary Fig. S3).

**Figure 2:**
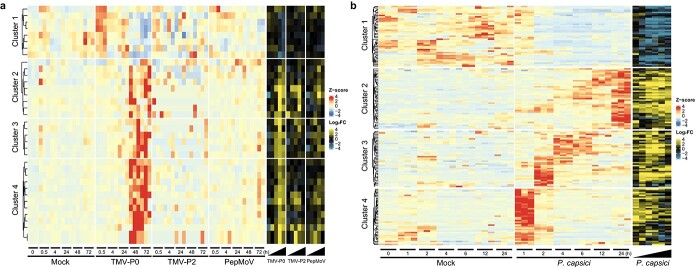
**Analysis of *CaRLP* expression patterns during pathogen infection.** (a) Heat map displaying the time-course expression profiles of *CaRLP*-differentially expressed genes (*CaRLP*-DEGs) identified in plants treated with TMV-P0, TMV-P2, and PepMoV. The left side (red to blue scale) and right side (yellow to blue scale) of the heatmap indicate DEGs with z-score and log_2_(fold-change; FC) values, respectively. (b) Heat map illustrating the time-course expression profiles of *CaRLP*-DEGs identified in plants treated with *P. capsici*.

Of the 438 *CaRLPs*, 35 were differentially expressed between TMV-P0-inoculated and control plants, and six were differentially expressed between PepMoV-inoculated and control plants (fold-change ≥2) at one or more time points (Supplementary Fig. S4 and Supplementary Table S6). However, no *CaRLP*-DEGs were identified between TMV-P2-inoculated and control plants (Supplementary Fig. S4). Heat map analysis divided the identified *CaRLP-*DEGs into four hierarchical clusters (Fig. 2a). In each cluster, *CaRLP*-DEGs identified between TMV-P0 vs. control treatments showed dynamic expression patterns, unlike those identified in TMV-P2 vs. control and PepMoV vs. control treatments (Fig. 2a and Supplementary Fig. S5a). Cluster 1 was enriched in *CaRLPs* down-regulated in TMV-P0- and PepMoV-infected plants at 72 h post-inoculation (hpi). *CaRLP*-DEGs in clusters 2, 3, and 4 were up-regulated at later time points, mainly in TMV-P0-inoculated plants.

Next, we compared the transcriptome of *P. capsici*-inoculated plants at 1, 2, 4, 6, 12, and 24 hpi with that of control plants and identified 158 *CaRLP*-DEGs (fold-change ≥2) at one or more time points (Fig. 2b and Supplementary Table S7). This number was much higher than that obtained from virus-inoculated plants. These 158 *CaRLPs* were also divided into four clusters by hierarchical clustering analysis (Fig. 2b and Supplementary Fig. S5b). *CaRLP*-DEGs present in cluster 1 were down-regulated, whereas those in cluster 2 were up-regulated at later time points. Genes in cluster 3 were highly expressed at 2, 4 and 6 hpi, but their expression decreased over time. DEGs in cluster 4 showed very strong up-regulation at 1 hpi and then down-regulation. Thirty-one CaRLPs-DEGs were identified in both virus- and *P. capsici*-inoculated plants, and they were referred to as common *CaRLP*-DEGs. Most of these 31 common *CaRLP*-DEGs showed increased expression over time in both virus- and *P. capsici*-inoculated plants and were classified into clusters 2, 3, and 4 in virus RNA-seq data and clusters 2 and 3 in *P. capsici* RNA-seq data (Fig. 2). Taken together, comprehensive transcriptome profiling suggests that CaRLPs are involved in an immune responses against biotic stresses, including viruses and oomycetes.

### Construction of comprehensive co-expression networks of *CaRLPs* using the RNA-seq data of pathogen-challenged pepper plants

**Figure 3:**
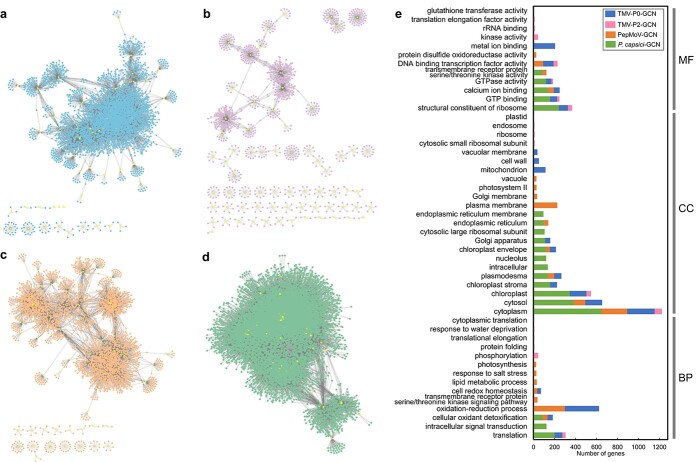
**Analysis of the co-expression network (GCN) of *CaRLPs* identified using the RNA-seq data of biotic stress-treated pepper plants.** (a–d) GCN comprising *CaRLP* hub genes identified in plants treated with TMV-P0 (a), TMV-P2 (b), PepMoV (c), and *P. capsici* (d). Yellow dots in the GCN indicate *CaRLPs*. (e) Top 20 GO terms significantly enriched in each of the four GCNs. The top 20 GO terms (*P* < 0.01) were selected in order of the largest number of genes from each of the four GCNs. BP, biological process; CC, cellular component; MF, molecular function.

To understand the functional implications of *CaRLPs* expressed during pathogen infection, CaRLP-targeted GCNs were constructed using all 102 RNA-seq datasets (described above). Four GCNs involving CaRLPs as hub genes were identified, one each from the RNA-seq data of TMV-P0-, TMV-P2-, PepMoV-, and *P. capsici*-challenged plants; these CaRLP-GCNs consisted of 4041 nodes with 11 825 edges, 1073 nodes with 1194 edges, 3732 nodes with 7933 edges, and 10 878 nodes with 84 255 edges, respectively (Fig. 3a, 3b, 3c, and 3d). Gene ontology (GO) enrichment analysis was performed for the modules in each of the GCNs identified. Various GO terms were enriched in the molecular function (MF), cellular component (CC), and biological process (BP) categories. Interestingly, two GO terms, “oxidation–reduction process” and “cellular oxidation detoxification,” were overrepresented in the BP category in the three pathogen-treated datasets of “CM334,” which showed a resistance response to TMV-P0, PepMoV, and *P. capsici* (Fig. 3e). These two biological processes are involved in the plant immune response: reduction–oxidation changes occur in response to pathogen invasion and are associated with the HR, a programmed execution of challenged plant cells [51]. Cellular oxidation detoxification has also been reported in plants under stress [52]. The GO term “phosphorylation” was enriched in the GCN from the TMV-P2 treated RNA-seq dataset. Previously, Wu et al. [53] reported that phosphorylation is induced following plant virus infection. RLPs associate with SOBIR1 and BAK1, and the phosphorylation cascade is triggered following activation of the RLP by ligand binding. PRR-derived signals are transmitted via a further phosphorylation cascade, including mitogen-activated protein kinases and calcium-dependent protein kinases, to downstream targets, such as the NADPH oxidase RBOHD, plasma membrane (PM)-resident H^+^-ATPases, and transcription factors (TFs) during PAMP-triggered immunity [54]. These findings suggest that CaRLPs and the corresponding genes in GCNs are involved in the biotic stress response in pepper. In addition, we performed Kyoto Encyclopedia of Genes and Genomes (KEGG) pathway analysis of genes belonging to each GCN. Pathway enrichment was associated with the plant immune response, such as “biosynthesis of antibiotics,” “phenylalanine metabolism,” and “phenylpropanoid biosynthesis” in TMV-P0-, PepMoV-, and *P. capsici*-specific CaRLP-GCNs, respectively (Supplementary Fig. S6). Taken together, GO and KEGG enrichment analyses of GCNs showed that genes connected with CaRLPs in GCNs are potentially involved in immune response in pepper.

SOBIR1 is essential to trigger the defense response by RLPs, and BAK1 is also required for RLP function [19]. To determine whether the co-receptors SOBIR1 and BAK1 are co-expressed with RLPs under various biotic stresses in pepper, we attempted to identify putative pepper orthologs of SOBIR1 and BAK1 in CaRLP-GCNs. Using SOBIR1 and BAK1 sequences from *Arabidopsis* and tomato, 2 and 8 pepper SOBIR1 and BAK1 orthologs were identified in the pepper genome (Supplementary Table S8). All 8 pepper orthologs of BAK1 were detected in *P. capsici*-CaRLP-GCN, 3 genes in TMV-P0-CaRLP-GCN, and 1 gene in PepMoV-CaRLP-GCN. No BAK1 orthologs were observed in TMV-P2-CaRLP-GCN. Two pepper SOBIR1 orthologs were detected in CaRLP-GCNs from *P. capsici*, TMV-P0, and PepMoV and one in TMV-P2. The putative pepper co-receptors were correlated with multiple CaRLPs in each GCN. These co-receptors with connected CaRLPs may be involved in the defense response against pathogens in pepper.

### Identification of biotic stress-responsive core *CaRLPs*

**Figure 4:**
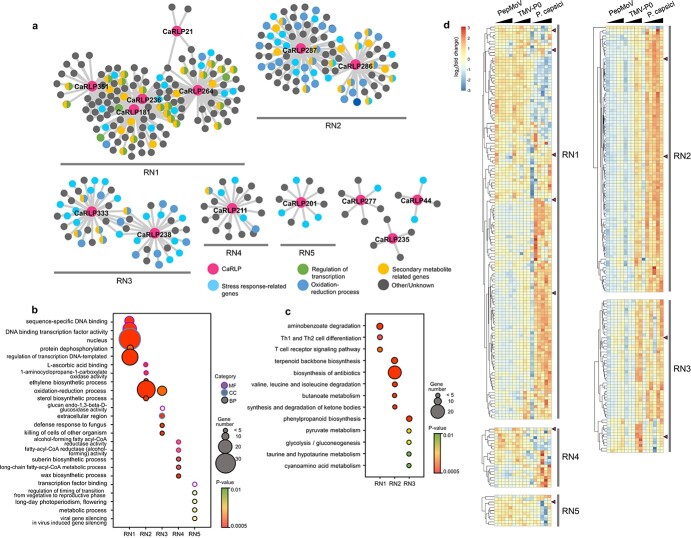
**Identification of biotic stress-responsive core *CaRLPs* in a universal GCN.** (a) Intersection of three GCNs of PepMoV-, TMV-P0-, and *P. capsici*-infected plants. The co-expression network modules containing more than 10 nodes were designated as RN1–RN5. Magenta nodes represent *CaRLPs*, and other colored nodes represent their annotated functions by GO analysis. (b) Top 5 GO categories enriched (*P* < 0.01) in five modules (RN1 to RN5). The Y-axis and X-axis in the bubble plot represent the GO category and different modules, respectively. The purple, blue, and black borders of the circle represent molecular function (MF), cellular component (CC), and biological process (BP), respectively. The size and color of each bubble represent the number of DEGs and *P*-value for each category, respectively. (c) Bubble plot showing the results of KEGG pathway-enriched analysis of five co-expression modules (RN1 to RN5). The Y-axis and X-axis in the bubble plot represent the enriched KEGG pathways and network modules, respectively. The size and color of each bubble represent the number of DEGs and *P*-value for each category, respectively. (d) Expression profiles of genes in RN1–RN5 modules. The expression values were normalized to the value of control samples (Mock). The module names are indicated on the right side of the heatmap. Magenta triangles on the right side of the heat map indicate hub *CaRLPs* in each module. Black triangles on the top of the heat map represent the time-course of each pathogen infection.

To identify *CaRLPs* involved in resistance to multiple pathogens, we merged the CaRLP-GCNs derived from the RNA-seq datasets of TMV-P0-, PepMoV-, and *P. capsici*-infected plants, thus constructing a universal resistance-responsive GCN (hereafter referred to as the RN). The RN contained eight modules (5 of 8 modules were named RN1–5 according to the module size), with a total of 14 hub *CaRLPs* (Fig. 4a).

Next, we performed GO and KEGG enrichment analyses and expression analysis to determine the biological processes and pathways affected during the plant immune responses of *CaRLPs* and associated pepper genes in the RN. Thus, we focused on GO terms belonging to the BP category in these modules. Stress-related genes were enriched in RN modules (Fig. 4a and 4b). Notably, genes involved in the stress response were enriched more in the RN2 and RN3 modules. GO terms related to plant defense mechanisms such as “oxidation–reduction process,” “sterol biosynthetic process,” and “ethylene biosynthetic process” were highly enriched in the RN2 module, while “oxidation–reduction process,” “defense response to fungus,” and “cellular oxidant detoxification” were highly enriched in RN3. As mentioned above, oxidation–reduction and cellular oxidant detoxification occur in plants in response to pathogen attacks. Most pathogenic fungi and oomycetes take up sterols from the external environment, most likely from the host cell membrane, during pathogenesis [55]. In addition, ethylene acts as a signaling molecule during stress [56]. The results of KEGG pathway analysis revealed the enrichment of defense-related pathways such as “biosynthesis of antibiotics,” “terpenoid backbone biosynthesis,” and “phenylpropanoid biosynthesis” in the RN (Fig. 4c). “Terpenoid backbone biosynthesis” and “phenylpropanoid biosynthesis” pathways produce secondary metabolites, which are involved in plant defense [57]. Taken together, these findings indicate that genes co-expressed with *CaRLPs* in the RNs are involved in the biotic stress response. We also examined the expression profiles of genes in the RN (Fig. 4d). Based on expression patterns, genes in RN1 were divided into two types: those highly up-regulated in response to both PepMoV and TMV-P0 and those up-regulated mainly in response to *P. capsici*. Expression of genes in the RN2 and RN3 modules increased over time in response to *P. capsici* and occurred at later time points in response to TMV-P0. In response to PepMoV, the genes were up-regulated with lower expression than during the TMV-P0 response but showed a similar expression pattern to those of TMV-P0 in both the RN2 and RN3 modules. The expression of genes in each module was significantly correlated, indicating these genes are tightly connected. These results suggest that hub *CaRLPs* in the RN play a role in the resistance to multiple biotic stresses.

### Functional validation of core *CaRLPs* involved in the HR-like response to pathogen invasion

We hypothesized that core *CaRLPs*—i.e. hub genes in a universal GCN—are involved in the resistance response to biotic stresses. To decipher the core *CaRLPs* of the GCN, which potentially function in the biotic stress response, we silenced or knocked-down gene expression of nine *CaRLP* genes in the pepper cultivar “Nockwang” using virus-induced gene silencing (VIGS). These nine genes included two genes not belonging to the RN (*CaRLP35* and *71*) and seven core *CaRLPs* belonging to the RN (*CaRLP181*, *211*, *264*, *277*, *286*, *287,* and *351*); the remaining 7 of 14 core *CaRLPs* were excluded from this analysis because their nucleotide sequence was none-specific for the VIGS assay. In addition, two CaRLPs not belonging to the RN were randomly selected from CaRLPs with gene-specific sequences for the VIGS assay. VIGS constructs were made by cloning a sequence unique to each of the nine *CaRLPs* into a *Tobacco rattle virus* (TRV) vector; notably, because *CaRLP286* and *287* exhibit high sequence similarity, these genes can be silenced using a single construct containing a sequence common to the two genes. The expression level of each *CaRLP* was significantly lower in *CaRLP*-silenced pepper plants than in the *TRV2-GFP* control (Supplementary Fig. S7), although no significant phenotypic difference was observed between *TRV2-CaRLP* and control plants (Supplementary Fig. S8), indicating that the eight *CaRLP* constructs did not affect the growth and development of pepper plants.

**Figure 5:**
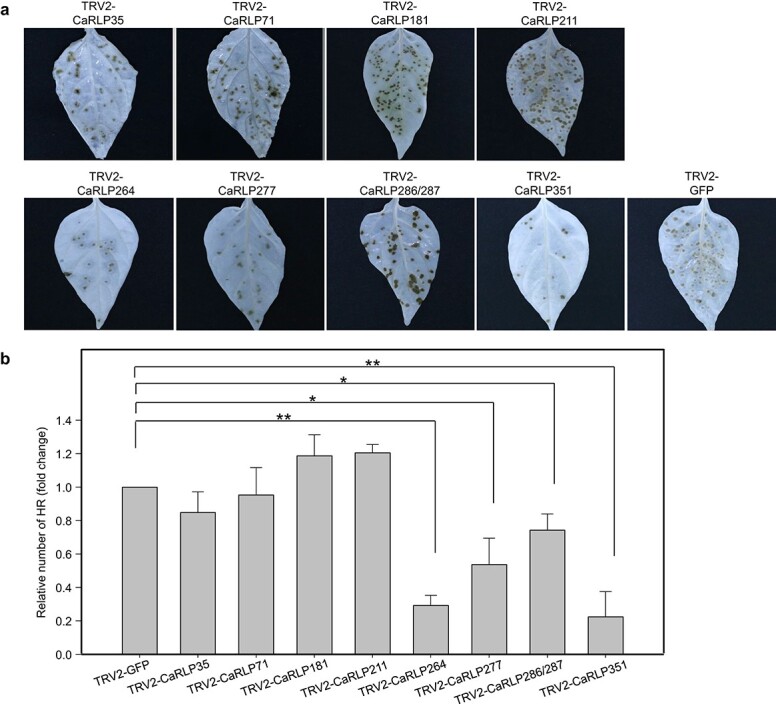
**Assessment of HR lesions in CaRLPs-silenced pepper plants following TMV-P0 infection.** (a) Photographs of TMV-P0-inoculated leaves of *CaRLP*-silenced plants. The photos were taken at 3 dpi. Chlorophyll was removed using ethyl alcohol. (b) Reduced HR lesion numbers in *CaRLP*-silenced plants inoculated with TMV-P0. The data are expressed as means ± standard error (SE) of three independent experiments (*n* = 24). Asterisks indicate statistically significant differences compared with the *TRV2:GFP* control (^*^p < 0.05, ^**^p < 0.01; Student’s *t*-test).

To investigate whether the silencing of *CaRLPs* affects HR, a form of programmed cell death (PCD), following TMV-P0 infection, we simultaneously inoculated *CaRLP*-silenced pepper plants and control plants with TMV-P0 and monitored their phenotypes. The number of HR lesions on TMV-P0-inoculated leaves was significantly lower in *TRV2-CaRLP264*, *−277*, *−286/287,* and *− 351* lines than in *TRV2-GFP* plants at 48 hpi (Fig. 5a). The level of HR in these four *CaRLP*-silenced lines was decreased by 0.22- to 0.72-fold compared with that in control plants (Fig. 5b). By contrast, the silencing of other *CaRLP* genes caused no significant change in the number of HR lesions. The data suggest that these *CaRLP* genes are involved in activating defense mechanisms and PCD following pathogen infection in pepper.

### Suppressed defense responses of core *CaRLP*-silenced pepper plants to various pathogens

To determine whether the core *CaRLPs* are involved in broad-spectrum resistance to various pathogens, we tested the response of *CaRLP*-silenced and *TRV2-GFP* control plants to *Xanthomonas axonopodis* pv. *glycines* 8ra (Xag8ra), *Ralstonia solanacearum* (Rsol), and *P. capsici*. We examined three different responses of *CaRLP*-silenced plants to the above-mentioned pathogens—non-host resistance to Xag8ra, host resistance to Rsol, and susceptible response to *P. capsici*. We selected the three *CaRLPs* (*CaRLP264*, *277,* and *351*) that showed the most significant differences in the resistance response to TMV-P0 infection (Fig. 5).

**Figure 6:**
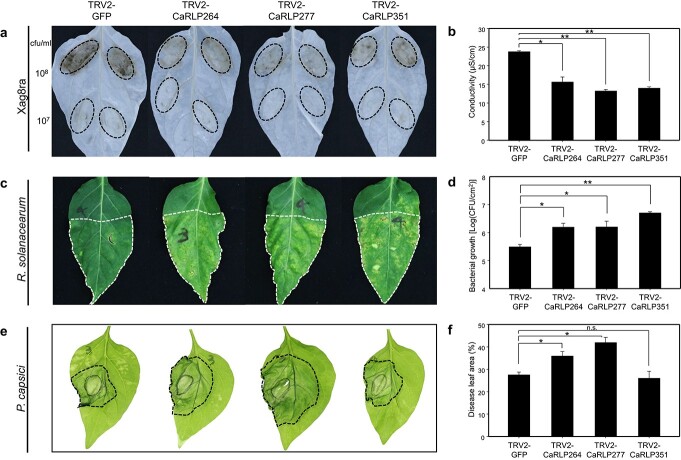
**Broad-spectrum resistance of core *CaRLP*-silenced peppers against various pathogens.** (a) Response to Xag8ra inoculation on leaves of *CaRLP*-silenced plants. The photos were taken at 2 dpi. Chlorophyll was removed using ethyl alcohol. The dotted lines on leaves indicate the inoculated area. (b) Ion leakage data of Xag8ra-inoculated leaves. Ion leakage was measured using leaf disks from the inoculation lesion of 10^8^ cfu/ml of Xag8ra. (c) Response of *Ralstonia solanacearum* inoculation on the leaves of *CaRLP*-silenced plants. The photos were taken at 8 dpi. The dotted lines on leaves indicate the inoculated area. (d) Bacterial growth of *R. solanacearum* in silenced plants. (e) Disease symptoms caused by *P. capsici* inoculation of the leaves of *CaRLP*-silenced plants and control plants. The photos were taken at 3 dpi. The dotted lines on leaves indicate disease lesions. (f) Disease lesion width normalized to the total leaf area. All the data are expressed as means ± SE from three independent experiments. Asterisks indicate statistically significant differences compared with the *TRV2:GFP* control (^*^p < 0.05, ^**^p < 0.01; Student’s *t*-test). n.s., not significant (*p* > 0.05).

To investigate the role of *CaRLPs* during the HR response of non-host resistance [49, 58], control plants (*TRV2-GFP*) and *CaRLP*-silenced plants (*TRV2-CaRLP264*, -*CaRLP277,* and -*CaRLP351*) were infiltrated with Xag8ra (10^8^ cfu/ml) (Fig. 6a). Xag8ra-inoculated plants of each *CaRLP*-silenced line showed significantly reduced HR-like cell death compared with control plants at 48 hpi. In addition, quantification of ion leakage from the inoculation-induced lesion showed that the conductivity of each *CaRLP*-silenced line was approximately 1.5- to 2-fold lower than that of control plants (Fig. 6b). This finding suggests that the core *CaRLPs* play a crucial role in HR-based immunity of pepper plants against Xag8ra.

Next, we performed leaf infiltration of Rsol, the causal agent of bacterial wilt disease, into *CaRLP*-silenced plants of the *C. annuum* cultivars “MC4” and “Subicho” which are resistant and susceptible to Rsol, respectively [59]. In the resistant cultivar (“MC4”), three *CaRLP*-silenced plants rapidly developed leaf wilting symptoms including necrosis and yellowing, whereas the control plants did not (Fig. 6c). Furthermore, the growth of Rsol was increased significantly by approximately 5- to 15-fold in *CaRLP*-silenced plants compared with that in control plants at 3 dpi (Fig. 6d). In the susceptible cultivar (“Subicho”), the growth of Rsol was also significantly increased in CaRLP-silenced plants compared with that in control plants (Supplementary Fig. S9). The bacterial growth of the control plants (GFP-silenced plants) in the susceptible cultivar showed no significant differences with that in CaRLP-silenced plants of the resistant cultivar. These findings suggest that silencing *CaRLP264*, *CaRLP277*, and *CaRLP351* enhances the susceptibility to Rsol.

Finally, the leaves of CaRLP-silenced and control plants were also challenged with *P. capsici*, and disease development was examined at 3 dpi. The *CaRLP264*- and *CaRLP277*-silenced plants showed larger disease lesions than control plants, whereas *CaRLP351*-silenced plants showed no significant difference in the lesion size compared with the control (Fig. 6e and 6f). This finding suggests that CaRLP263 and CaRLP277 are involved in the defense response to *P. capsici*. Taken together, plants of each *CaRLP*-silenced line consistently showed significant suppression of broad-spectrum defense against plant pathogens, including viruses, bacteria, and oomycetes. Overall, our data suggest that core *CaRLPs* of the universal GCN are involved in the resistance against multiple biotic stresses. Thus, these *CaRLPs* can be used to engineer cultivars with broad-spectrum resistance against diverse pathogens.

## Discussion

Plants sense pathogens via both cell surface and intracellular receptors. RLPs represent the primary layer of defense against pathogen infection in the innate immune system. In the present study, we identified many *CaRLP* genes in the pepper genome and selected variable biotic stress-responsive *CaRLP* genes as components of GCNs using 102 RNA-seq datasets. We demonstrated that three hub *CaRLPs* in the universal GCN are involved in broad-spectrum resistance against diverse pathogens.

Many genes in eukaryotic genomes are organized in clusters of various sizes and gene densities. Clusters containing resistance gene analogs (RGAs), including NLR, RLKs, and RLPs, have been reported in plants [26, 46, 60]. In tomato and pepper, several genes related to RGAs are localized in clusters on various chromosomes [50, 60, 61]. Consistent with this data, we observed that *CaRLPs* belonging to the same phylogenetic group were mostly located in the same cluster; thus, they showed uneven chromosomal distribution (Fig. 1). Information on the chromosomal location of *CaRLPs* would be highly valuable for the identification of functional RGAs.

GCNs could provide important clues to characterize novel genes, based on the analysis of potentially functionally associated co-expressed genes, using large-scale gene expression datasets [30]. Here, we attempted to infer the function of *CaRLPs* under various biotic stresses by analyzing GCNs derived from numerous RNA-seq datasets of *C. annuum* “CM334.” GCNs were constructed using *CaRLPs* as hub genes in two steps: the construction of large CaRLP-GCNs, based on the RNA-seq of each biotic stress, and the construction of the intersection of CaRLP-GCNs, according to the type of biotic stress. Four large CaRLP-GCNs were constructed, each corresponding to four biotic stresses and containing 1073–10 878 genes. These CaRLP-GCNs showed that *CaRLPs* are co-expressed with numerous other pepper genes under various biotic stresses. This result is consistent with previous studies showing that the plant response to pathogens is extensively regulated at the transcriptional level [62–64].

The intersection of three GCNs led to the construction of the RN (Fig. 3 and Fig. 4). The RNA-seq data in this study was obtained from *C. annuum* cultivar “CM334,” which is resistant to TMV-P0, PepMoV, and *P. capsici* but susceptible to TMV-P2. Consequently, GO enrichment analysis of genes in the RN revealed the enrichment of various stress-related terms such as “oxidation–reduction process,” “defense response to fungus,” “response to biotic stimulus,” and “cellular response to oxidative stress” (Fig. 4). In addition, numerous genes were enriched not only in stress-related GO terms but also in transcriptional regulation (Fig. 4). Most of the genes in the RN enriched under “regulation of transcription” encoded TFs, such as WRKY, AP2/ERF domain-containing proteins. These TFs play critical roles in abiotic and biotic stresses [65]. For example, WRKY proteins are involved in the RLP-mediated defense response. Signal transduction of AtRLP51 is mediated by BDA1, an ankyrin-repeat-containing protein with four transmembrane domains, to provoke the plant defense response by activating WRKY70 [66, 67]. Taken together, these data suggest that *CaRLPs* in universal GCNs are co-regulated with TFs under biotic stress.

We hypothesized that core *CaRLPs* in the universal GCN (RN) are involved in the response to biotic stresses. To test our hypothesis, we characterized the function of core *CaRLPs* using VIGS. We could not develop stable transgenic plants in pepper because of the limitation of the transgenic system for pepper and low regeneration rate of pepper plants under in vitro conditions [68]. Of the six *CaRLPs* tested in this study, plants silenced for the expression of three *CaRLPs* showed reduced HR-like cell death following TMV-P0 and Xag8ra inoculation (Figs. 5 and 6). The silencing of each *CaRLP* significantly enhanced the disease susceptibility of pepper plants to Rsol and *P. capsici* compared with control plants (Fig. 6 and Supplementary Fig. S9). These data suggest that the core *CaRLPs* in the universal GCN induce broad-spectrum resistance against plant pathogens. Thus, the construction of a universal GCN from comprehensive transcriptome datasets could provide useful clues to uncover the roles of genes in various biological processes.

Resistance gene-mediated immunity is a highly effective immune system against specific pathogens. However, PRRs, located on the plant cell surface, confer resistance to many pathogens. In previous studies, few plant PRRs showed broad-spectrum resistance to pathogens. Expression of the *Arabidopsis* elongation factor Tu (EF-Tu), one of the PRRs, in *N. benthamiana* and tomato increases resistance to *Pseudomonas*, *Agrobacterium*, *Xanthomonas,* and *Ralstonia* [5]. In potato, the elicitin response (ELR) receptor-like protein associates with the immune co-receptor BAK/SERK3 and mediates broad-spectrum recognition of elicitin proteins from several *Phytophthora* species [4]. In addition, the suppression of the pepper lectin receptor kinase gene *CaLecRK-S.5*, which acts as a PRR, shows enhanced susceptibility to PepMoV, *Xanthomonas*, and *P. capsici* [69]. In this study, by generating a conserved GCN, we identified PRRs involved in broad-spectrum resistance against diverse plant pathogens. Three *CaRLPs* (*CaRLP264*, *277,* and *351*) enhanced susceptibility to TMV-P0, *Xanthomonas*, *Ralstonia*, and *P. capsici* (Fig. 5 and Fig. 6). Thus, these *CaRLPs* may be used to develop Solanaceae crop cultivars with broad-spectrum resistance against diverse pathogens. Overall, a universal GCN with comprehensive RNA-seq datasets can provide key insights to unveil gene functions in biological processes.

## Materials and methods

### Identification of *CaRLP* genes

Thirteen characterized plant *RLP* genes (Supplementary Table S9) were used to obtain *CaRLP* gene sequences, which were used to build a hidden Markov model (HMM) domain with the HMMER software package (version 3.0; http://hmmer.org/), and identified putative RLP-encoding genes against the *C. annuum* “CM334” v. 1.55 genome. Next, tBLASTn searches were performed using the HMMER domain from amino acid sequences encoded by the pepper genome (threshold: 10^−4^). Consequently, 600–750 hits to genes in the pepper genome were obtained from the BLAST output, accounting for 7376 genes in total. This gene set was processed to remove redundant sequences by manual curation, thus obtaining 784 non-redundant candidate genes. The structure of CaRLPs was annotated using Pfam [70] and SMART [71] databases, and genes with kinase and NB-ARC domains were filtered out using Pfam IDs PF07714.12, PF00069.20 and PF00931. Finally, 438 CaRLPs were identified from the “CM334” genome. The subcellular localization and prediction of transmembrane helices of CaRLP proteins were performed using TargetP-2.0 (http://www.cbs.dtu.dk/services/TargetP/) and TMHMM ver2.0 (http://www.cbs.dtu.dk/services/TMHMM/), respectively.

### Phylogenetic analysis and classification of CaRLPs

The CaRLPs were classified based on the results of phylogenetic analysis and sequence similarity-based clustering, as described previously [26, 46]. Clustering analysis of full-length amino acid sequences of *Arabidopsis*, tomato, pepper and reported RLPs was performed using OrthoMCL [72]. RLPs within the same cluster were determined to be identical subgroups to the phylogenetic subgroups. RLPs clustered as singletons (mostly partial and short sequences) were identified using a BLASTP search against identified RLPs, and subgroups were assigned.

A conserved domain of an HMM profile was built based on the amino acid sequence of the conserved C3-D region [27] of known RLPs. A phylogenetic tree was constructed based on the C3-D domain of 56 AtRLPs [24, 27], 176 SlRLPs [26], 438 CaRLPs, and 13 RLPs reported by HMM search (E-value <0.001). *RLP* genes containing less than 80% of the full-length C3-D domain sequence were excluded. Multiple sequence alignment of the C3-D domains of RLPs was performed using MUSCLE (http://www.ebi.ac.uk/Tools/msa/muscle/). The alignment result was used to build a phylogenetic tree using PhyML (http://www.phylogeny.fr/), with default parameters (SH-like approximate likelihood-ratio test for branch support), and the resulting phylogenetic trees were edited using the MEGA8 software (http://www.megasoftware.net/).

### Chromosomal location, physical cluster, and motif analyses

The chromosomal location of *CaRLPs* was determined based on the genome sequence of the pepper cultivar “CM334” [44]. MapChart [73] was used to draw the location of RLPs on chromosomes. Physical clustering of *CaRLPs* in the pepper genome was determined based on two criteria: 1) the gene cluster spans a region of 200 kb or less; 2) the cluster contains fewer than eight non-*RLP* genes between two *CaRLPs* [26, 46].

Conserved motifs in CaRLPs were identified using the MEME suite (http://meme-suite.org/tools/meme), with default settings except for the following parameters: maximum number of motifs, 20; minimum width of motifs, 15; maximum width of motifs, 200. Subsequently, MAST (http://meme-suite.org/tools/mast) was performed using datasets including the protein sequences of CaRLPs and known RLPs with default E-values.

### RNA-seq library construction

Changes in the expression profiles of *CaRLPs* following *P. capsici* infection were investigated in the pepper cultivar “CM334” by RNA-seq analysis. The leaves of 4- to 5-week-old pepper plants were infiltrated with *P. capsici* (5 × 10^4^ zoospore/ml), and infected leaves were collected at 0, 1, 2, 4, 6, 12, and 24 hpi in three biological replicates. Total RNA was isolated using the TRIzol Reagent (Invitrogen, Carlsbad, CA, USA), according to the manufacturer’s instructions. RNA-seq libraries were constructed as described previously [47]. All 39 RNA-seq libraries (21 libraries of *P. capsici*-infected samples and 18 libraries of control samples) were sequenced using Illumina HiSeq 2000 (Illumina Inc., San Diego, CA, USA).

### RNA-seq data analysis

Quality control of RNA-seq data of *P. capsici*-infected samples was performed by removing low-quality reads and possible contaminants, as described previously [42, 43]. Adapter and low-quality sequences were filtered using Cutadapt [74] and Trimmomatic [75], based on the Phred quality threshold of 20. In addition, the transcriptome data of pepper plants infected by TMV-P0, TMV-P2, and PepMoV were obtained from previous studies [45, 47] to analyze the expression of *CaRLPs*.

### Gene expression analysis

The expression profiles of *CaRLPs* under biotic stresses were analyzed using the RNA-seq data of TMV-P0-, TMV-P2- and PepMoV-inoculated pepper plants and that of *P. capsici*-inoculated pepper plants. Sequence reads from all RNA-seq datasets were aligned to the “CM334” reference genome using Hisat2 [76]. Filtered clean reads of virus RNA-seq and *P. capsici* RNA-seq were normalized to reads per kilobase per million mapped reads (RPKM) and fragments per kilobase per million mapped fragments (FPKM), respectively. PCA was conducted to elucidate the dynamics of global gene expression and examine sample variation. Average log_2_-transformed RPKM and FPKM values for the virus and *P. capsici* groups, respectively, were used for PCA with a previously published code with modification [42]. DEGs were identified using the DESeq2 package (FDR < 0.05) [77]. The expression patterns of DEGs were visualized using ComplexHeatmap [78].

## GCN construction and GO and KEGG enrichment analyses

The GCN was constructed from 102 RNA-seq datasets using the exp2net function of the mlDNA package [79], and inferred using the Pearson’s product moment correlation coefficient at a significance level of *P* < 0.01. In the next step, genes co-expressed with *CaRLPs* were identified by filtering the correlation coefficient (|r| > 0.8) and only directed interaction. The GCN was visualized using Cytoscape v3.4.0 [80]. To identify gene networks involved in different stress responses, GCNs containing *CaRLP* genes were extracted by different combinations of all stresses using Merge Tools in Cytoscape. GO and KEGG enrichment analyses were performed using GOseq [81] in R packages using Pepper v. 1.55 genome annotation from BLAST2GO [82]. To identify the pepper orthologs of BAK1 and SOBIR1, BLAST was performed using AtBAK1 (AT4G33430), AtSOBIR1 (AT2G31880), SlBAK1 (Solyc10g047140.2.1), and SlSOBIR1 (Solyc06g071810.1.1) sequences against the *C. annuum* “CM334” v. 1.55 genome (Supplementary Table S8).

### VIGS

Pepper cultivars *C. annuum* “Nockwang,” “MC4,” and “Subicho” were used to analyze the effect of *CaRLP* gene silencing on the defense response. Seedlings with two fully expanded cotyledons were used for the VIGS assay. The 3′ or 5′ untranslated region (UTR) with the CDS region of eight *CaRLP* genes (*CaRLP35*, *CaRLP71*, *CaRLP181*, *CaRLP211*, *CaRLP264*, *CaRLP277*, *CaRLP286*/*287* and *CaRLP351*) was amplified and cloned into the pTRV2 vector. The resulting pTRV2-CaRLP constructs were transformed into the *Agrobacterium tumefaciens* strain GV3101. VIGS was conducted as described previously [83]. Plants infiltrated with pTRV2-GFP or pTRV2-PDS with pTRV1 were used as a control. One leaf was harvested from each *CaRLP*-silenced plant to extract RNA and measure the silencing efficiency.

### Pathogen inoculation

All pathogen inoculations were performed on the 3^rd^ and 4^th^ true leaves of *CaRLP*-silenced and control pepper plants 4–5 weeks after the VIGS assay. Plants were challenged with three different types of pathogens—virus (TMV-P0), bacteria (Xag8ra and Rsol) and oomycete (*P. capsici*). The TMV-P0 inoculum was prepared from 1 g of infected *N. benthamiana* leaves using 10 ml of 0.1 M phosphate buffer (pH 7.0). TMV-inoculated leaves were monitored and harvested at 3 days post-inoculation (dpi). To assess the formation of lesions on TMV-inoculated leaves, chlorophyll was removed using ethyl alcohol. To conduct the Rsol-response assay, Rsol “SL1931” was cultured first in TZC agar medium at 28°C for 2 days and then in CPG medium at 28°C for 24 h, and then suspended in distilled sterile water. The Rsol suspension was diluted to a concentration of 10^5^ cfu/ml and infiltrated into the leaves of *CaRLP*-silenced pepper plants. Subsequently, Rsol-inoculated plants were grown in a growth chamber at 28 ± 2°C, 70% relative humidity and a 16-h light/8-h dark photoperiod, and inoculated leaves were harvested at 5 dpi. Inocula of Xag8ra and *P. capsici* were prepared as described previously [49, 83]. The Xag8ra culture was suspended in 10 mM MgCl_2_ and then diluted to 10^7^ and 10^8^ cfu/ml. The Xag8ra-inoculated leaves were harvested at 2 dpi and used to measure conductivity and detect cell death. To prepare the *P. capsici* inoculum, the released zoospores were collected and diluted in distilled sterile water to a concentration of 1 × 10^5^ spores/ml. The *P. capsici* suspension was infiltrated into the leaves of *CaRLP*-silenced pepper plants and harvested at 3 dpi. All pathogen inoculations were conducted in at least three independent experiments, with 8–12 plants for per experiment.

### Quantification of ion leakage

Ion leakage from Xag8ra-inoculated leaves was measured as described previously [49]. Sixteen leaf disks (each 1 cm in diameter) were excised from 4–6 plants of each *CaRLP*-silenced line and floated on 15 ml of sterile distilled water for 2 h at room temperature. Next, the electrolyte leakage from leaf discs was measured using a conductivity meter (Eutech con 510; Thermo Scientific, Waltham, MA, USA).

### Bacterial cell counting

In Rsol-inoculated pepper plants, bacterial cell growth was measured, as described previously [59], with slight modifications. Six leaf disks (each 1 cm in diameter) were excised from the Rsol-inoculated leaves of 3–4 plants of each *CaRLP*-silenced line at 5 dpi. The leaf disks were ground in sterile distilled water, and serial dilutions were plated on CPG agar medium supplemented with gentamycin. The plates were incubated at 28°C, and bacterial cells were counted after 2 days.

## Supplementary Material

Web_Material_uhab003

## Data Availability

The data generated herein to support the results of this study are presented in the paper and its Supplementary Information files. Moreover, the generated and analyzed datasets of this study are available from the corresponding authors upon request.
